# Wild chimpanzees are infected with homologous types of human malaria

**DOI:** 10.1186/1475-2875-9-S2-O21

**Published:** 2010-10-20

**Authors:** Marco Kaiser, Dorothy H Bray, Zinta Zommers, Emmanuel Couacy-Hymann, Thomas R Gillespie, Heinz Ellerbrok, Fabian H Leendertz

**Affiliations:** 1 Centre for Biological Safety-1; Robert Koch-Institute, D-13353 Berlin-Wedding, Germany; 2 GenExpress GmbH, D-1 2103 Berlin, Germany; 3 ImmunoClin Ltd, London N12 8PE, UK; 4 Wildlife Conservation Research Unit, Recanati-Kaplan Centre, Department of Zoology, University of Oxford, Tubney OX13 5QL, UK; 5 LANADA/ LCPA, BP 206, Bingerville, Ivory Coast; 6 Department of Environmental Studies and Program in Population Biology, Ecology and Evolution, Emory University, Atlanta 30322, Georgia, USA; 7 Department of Environmental and Occupational Health, Rollins School of Public Health, Emory University, Atlanta 30322, Georgia, USA; 8 Research Group Emerging Zoonoses; Robert Koch-Institute, D-13353 Berlin-Wedding, Germany; 9 Department of Primatology, Max-Planck-Institute for Evolutionary Anthropology, 04103 Leipzig, Germany

## Background

Despite ongoing - and in some regions escalating - morbidity and mortality associated with malaria parasites, evolutionary epidemiology of *Plasmodium* is not well characterized. Recent studies using molecular approaches demonstrated that wild and captive gorillas and captive bonobos and chimpanzees are infected with *P. falciparum* and that these apes harbor parasites broadly related to *P. falciparum*[1]. Captive chimpanzees and bonobos had malaria parasites related to human *P. ovale* and *P. malariae* and various monkeys and one semi-wild chimpanzee had *P. vivax*[2]. It is not clear whether these apes harbor naturally these parasites or whether they are transmitted from humans. Most of the examined animals had close contact with humans, comparable studies in wild living apes are missing. We provide the first survey of *Plasmodium* diversity in wild chimpanzees living in an undisturbed tropical rainforest in Africa.

## Methods

We examined tissue from 16 wild West African chimpanzees that lived in the Tai National Park, Ivory Coast, where human contact with animals is limited to researchers who access the territory only during the day. Samples were collected from animals that died primarily from anthrax or respiratory disease. Generic real time PCR assay was used to detect all known *Plasmodium species.*

## Results

11/16 (68%) animals tested positive. Sequence analyses of cytB and 18S rRNA genes identified *P. malariae, P. ovale, P. vivax, P. gaboni, P. reichenowi, P. billcollinsi and P. billbraii.*

## Discussion

Previous examination of the role of our closest phylogenetic relatives, the great apes, in the evolution and persistence of human malarias has been limited by a lack of data from wild ape populations. Interpretation of patterns of malaria infection in captive ape populations must consider ample opportunities for human to ape transmission, negating the opportunity to investigate the evolutionary origins and public health-related risks of these parasites. Our examination of malaria parasites in wild chimpanzees demonstrates that these apes are most likely naturally infected with *Plasmodium species* homologous to *P. malariae, P. vivax* and *P. ovale* as well as *P. falciparum.* Whether wild great apes are the origin of these malaria types requires further investigation but they may act as reservoir of infection. These results have important implications for global efforts underway to eradicate malaria in humans including vaccine development based on animal variants of human parasites.
